# Global Patterns of Diversity and Selection in Human Tyrosinase Gene

**DOI:** 10.1371/journal.pone.0074307

**Published:** 2013-09-11

**Authors:** Georgi Hudjashov, Richard Villems, Toomas Kivisild

**Affiliations:** 1 Evolutionary Biology Group, Estonian Biocentre, Tartu, Estonia; 2 Department of Evolutionary Biology, Institute of Molecular and Cell Biology, University of Tartu, Tartu, Estonia; 3 Estonian Academy of Sciences, Tallinn, Estonia; 4 Division of Biological Anthropology, University of Cambridge, Cambridge, United Kingdom; University of Wisconsin, United States of America

## Abstract

Global variation in skin pigmentation is one of the most striking examples of environmental adaptation in humans. More than two hundred loci have been identified as candidate genes in model organisms and a few tens of these have been found to be significantly associated with human skin pigmentation in genome-wide association studies. However, the evolutionary history of different pigmentation genes is rather complex: some loci have been subjected to strong positive selection, while others evolved under the relaxation of functional constraints in low UV environment. Here we report the results of a global study of the human tyrosinase gene, which is one of the key enzymes in melanin production, to assess the role of its variation in the evolution of skin pigmentation differences among human populations. We observe a higher rate of non-synonymous polymorphisms in the European sample consistent with the relaxation of selective constraints. A similar pattern was previously observed in the *MC1R* gene and concurs with UV radiation-driven model of skin color evolution by which mutations leading to lower melanin levels and decreased photoprotection are subject to purifying selection at low latitudes while being tolerated or even favored at higher latitudes because they facilitate UV-dependent vitamin D production. Our coalescent date estimates suggest that the non-synonymous variants, which are frequent in Europe and North Africa, are recent and have emerged after the separation of East and West Eurasian populations.

## Introduction

Modern humans are genetically less diverse than other living hominoids. Most of human genetic diversity is found within rather than among populations. However, exceptions to this pattern can be found in genes that have been affected by natural selection. One of the most striking phenotypic differences among humans is in skin color. The degree of among population variation of this trait has been estimated as 88%, which is high compared to roughly 10-15% observed for genetic loci on average. Such high phenotypic differentiation is understood to be due to the effect of natural selection [1]. The geographical pattern of variation in human skin pigmentation correlates with the latitudinal differences in annual UV radiation (UVR) level [2]. Our skin color is defined by the amount, intracellular distribution and turnover of melanins – a mixture of dark photoprotective eumelanin and light pheomelanin [3]. Several evolutionary mechanisms that are dependent on UV level have been proposed to explain the variation in human skin pigmentation. These include the vitamin D [4], protection from UV-induced folate photolysis [5], sexual selection [6], skin cancer [3] and xeric stress hypothesis [7]. The most well established explanation assumes that the optimal degree of skin pigmentation is a balance between skin dark enough to protect our cells from UV radiation, yet light enough to permit sufficient vitamin D production. Increased concentration of protective eumelanin is essential to avoid damaging sunburns and extensive folate photolysis in (near-) equatorial areas, while it could be harmful in regions with low annual UV level due to reduced UV-dependent vitamin D synthesis [3,8].

Around 170 pigmentation genes with human orthologs have been identified in mice thus far [9]. Yet, only a small fraction of these candidate genes, including *TYRP1, OCA2, SLC45A2, SLC24A5, MC1R* and *KITLG*, are known to influence normal skin color variation in our species [10,11]. The most striking examples of single substitutional changes explaining a substantial proportion of phenotype variation of skin and hair color are: a non-synonymous polymorphism in the *SLC24A5* gene coding for a putative melanosomal cation exchanger and arguably responsible for up to 38% difference between European *vs.* African melanin index of the skin [12], a mutation in the *TYRP1* gene coding for tyrosinase-related protein 1 and accounting for the blonde hair in the Melanesian population [13], and variation in the *MC1R* gene coding for a G-protein coupled receptor and connected to the red hair and fair skin phenotype in Europeans and, possibly, Neanderthals [14,15]. The direction and nature of natural selection acting upon pigmentation loci is rather complex: some genes, including *SLC24A5*, *SLC45A2* and *KITLG*, have been found to be enriched for signals of positive selection [16–19]. On the contrary, the *MC1R* gene has an excess of non-synonymous mutations in Europe and East Asia and shows traces of either relaxed evolution under the absence of strong functional constraints or even selective enhancement of genetic diversity in low UV environment [15,20]. However, relatively narrow methodological capability for detection of ancient selection processes limits our knowledge about both spatial and temporal aspects of skin color evolution on the global level and leaves open the possibility that many more pigmentation genes may have been affected by selection in anatomically modern humans than recognized at the present moment.

The evolutionary history of human skin pigmentation is complex and may have involved several independent episodes of natural selection acting on different genes at different time points. Firstly, it is likely that pigmentation genes were affected by selection in the common ancestors of all living humans after they lost the protective fur [21] and split from other archaic hominins [22]. Secondly, selection for lighter skin acting upon the *KITLG* gene may have been invoked in proto-Eurasian population moving away from the UVR-rich equator after the African exodus [16,19,21,23–25]. And, thirdly, selection on vitamin D synthesis or sexual selection may have been an important evolutionary driver during the settlement of low UVR environments after the split of proto-European and proto-East Asian populations. Several lines of evidence point to convergent evolution in that last stage. While the East Asians display high frequencies of population-specific alleles in *OCA2, DRD2, DCT, ADAM17, ADAMTS20* and some other pigmentation candidate genes, the signals for selective sweeps in West Eurasia are centered on a different set of genes – *SLC24A5*, *SLC45A2* and *TYRP1*. The European-specific alleles of these genes have reached high frequencies over the past 19,000 to 11,000 years BP and have likely contributed to the evolution of light skin in Europe [10,11,21,23,25–29].

Human tyrosinase is one of the main rate-limiting enzymes in melanogenesis, catalyzing the first two steps, and at least one subsequent step, in the conversion of tyrosine to melanin [30]. The transmembrane protein (529 amino-acids) is coded by the *TYR* gene (11q14.3, MIM 606933), which has five exons and spans about 118 kb in total. Several SNP genotyping studies have suggested the role of this gene in normal variation of skin pigmentation. One *TYR* non-synonymous polymorphism, rs1042602 (Ser192Tyr), has been associated with differences between lightly and darkly pigmented individuals from South Asia [31]. The derived allele of this SNP has specifically high frequency in Europe where it is associated with eye color, freckles and skin pigmentation [29,32–34], and also with the risk of skin cancer [35–37]. However, the results of scans for natural selection on this gene have been inconsistent: a few studies have detected a weak signal of selection [27,29], while others have not been able to reject the neutral hypothesis [17–19,25,38,39]. The comparison of re-sequencing-based neutrality statistics from exon 1 and 2.8 kb of the 5’ flanking region with a genome-wide haplotype-based test also gave contradicting results: European and West African Senegalese populations showed significant deviation from neutrality in sequence-, but not in haplotype-based tests [28]. Moreover, recent comparison of five other pigmentation genes (*OCA2, TYRP1, DCT, KITLG* and *SLC45A2*) also revealed conflicting results with regards to evidence of positive selection detected from haplotype and re-sequencing data. Only in the case of the *SLC45A2* gene was the signal of positive selection from haplotype-based statistics unambiguously confirmed in tests based on re-sequencing data [40].

Here we will assess the extent of genetic variability and describe the geographic pattern of sequence variation of the human tyrosinase gene and its promoter region in a global sample. We will also address the question of the role of natural selection in shaping this genetic variation and its implications on normal skin color variation, using classical Tajima’s D sequence-based statistics, iHS and XP-EHH haplotype-based tests and also genome-wide F_ST_ population differentiation estimates.

## Materials and Methods

### Samples

Three overlapping datasets were generated and used in the current study:

1 Sequencing of the tyrosinase gene (*TYR*) was performed on DNA samples from 81 individuals representing genetic variation in 6 regions worldwide: Africa (n=24), Europe (n=16), East Asia (n=17), South Asia (n=13), Oceania (n=7) and America (n=4). Of these, 70 samples were from the HGDP-CEPH Human Genome Diversity Cell Line Panel [41]. The remaining eleven samples were healthy volunteers from South Asia and Europe. Samples were chosen to cover major continental regions in the HGDP-CEPH panel and additional individuals from India were sequenced to provide better representation of genetic diversity in South Asia.
2 Illumina genotyping data for 351 samples was used for genome-wide scans of positive selection: 338 samples were from previously published data [42-45], and 13 are reported here for the first time. Of the 351 samples, 70 were also involved in the re-sequencing project. Genotyped samples were divided into the same six (sub-) continental groups: Africa (n=59), Europe (n=68), East Asia (n=80), South Asia (n=83), Oceania (n=28) and America (n=33). An additional Bantu sample (n=19) from the HGDP-CEPH panel was used as a reference population in XP-EHH analyses.
3 The ancestral recombination graph was built using a combined set of 1108 individuals, including all the samples from panel 1 and 2 (362 samples in total), plus additional 746 HGDP-CEPH Illumina genotypes [42]. Samples were divided into seven groups according to their geographic origin: sub-Saharan Africa (n=123), North Africa (n=30), Europe (n=309), East Asia (n=246), South Asia (n=256), Oceania (n=36) and America (n=108).

Additional information on all samples used in the present study can be found in Table S1. DNA samples introduced for the first time in this study were collected with written informed consent. The study has been approved by the Bioethics Committee of the Estonian Biocentre.

### DNA sequencing and genotyping

The following regions of *TYR* were sequenced: the 5’ and 3’ flanking regions, five complete exons including exon-intron junctions, and additional 12 intronic regions. The total amount of sequence produced is about 24.3 kb: 12.9 kb and 0.4 kb come from the 5’ and 3’ flanking regions, respectively, 1.6 kb are coding sequences and 9.4 kb are intronic sequences. A graphical overview of the sequenced regions is available in Figure S1 and additional detailed information is presented in Table S2. PCR conditions and primer information are available upon request from the authors. Nucleotide sequences are available through GenBank, accession numbers KC201427-KC201588.

Aligned sequences were compared to the hg19, GRCh37 human reference sequence and chimpanzee genome sequence build 3.1. Individual haplotypes were further reconstructed using the PHASE algorithm [46,47] implemented in the DNASP 5.10 software package [48]. The phase of the singletons was, where possible, determined by a cloning procedure with CloneJET™ PCR Cloning Kit (Thermo Fisher Scientific Inc.).

New samples were genotyped using the HumanHap650Y BeadChip, while some of the already published samples were genotyped either by the same chip or using the Illumina Human610-Quad BeadChip (see Table S1 for details). The genotype data were processed and filtered with PLINK v 1.06 [49]. Our original master-file included only SNPs shared between the three Illumina genotyping arrays: HumanHap650Y, Human610-Quad and Human660W-Quad. We further excluded all SNPs with call rates less than 0.95. A total of 531,315 autosomal SNPs passed this criterion. We further applied additional filters, the filter choice depended upon the analysis performed: (a) for the whole-genome *d*_*i*_ we used autosomal non-phased genotypes with call rates equal to 1.0, and SNPs with minor allele frequency over 0.05; (b) for iHS and XP-EHH, first, the haplotype reconstruction was performed using BEAGLE 3.1 [50], and later the same filters as for the whole-genome *d*_*i*_ were applied to the phased data. After all filtering steps our non-phased dataset included 415,334 and phased dataset 491,253 autosomal markers.

### Sequence data-based tests

The Jukes-Cantor corrected nucleotide diversity (π) and Tajima’s D value [51] were estimated using the DNASP 5.10 software [48]. The significance of Tajima’s D statistics was assessed by two different approaches. Firstly, empirical *TYR* values were compared to 10,000 coalescent simulations performed under the hypothesis of selective neutrality and population equilibrium in ARLEQUIN 3.5.1.3 [52]. Secondly, Tajima’s D estimates of the longest consecutive sequence block (*ca* 13.8 kb), which comprises the 5’ flanking region and the first exon of *TYR* (Figure S1 and Table S2), were further compared to the average of D calculated from 10,000 neutral simulations using the COSI 1.2.1 software under the "best-fit" model [53]. COSI has been calibrated to simulate extant human genetic variation under neutrality. African, European and Asian sequences were generated using the parameters of the "best-fit" model. However, these parameters were not provided for American and Oceanian populations, for which we have chosen the closest proxy in terms of genetic similarity, namely the Asian model [42,54]. The model parameters were further adjusted to meet our re-sequencing project details: this included sequence length and mutation rate. Local *TYR* recombination rate from the HapMap GRCh37 genetic map was used in the coalescent simulations [55].

Within population differentiation was estimated using analysis of molecular variance (AMOVA) with ARLEQUIN 3.5.1.3 [52]; statistical significance was assessed by 1,000 permutations.

### Genotype data-based selection tests

The following whole-genome selection scans were performed: integrated haplotype score (iHS) [18], cross-population extended haplotype homozygosity (XP-EHH) [17] and pairwise F_ST_-based *d*_*i*_ [56]. All statistics were calculated using our Illumina genotyping sample set (n=351) for each of the six population groups separately. The dataset was filtered as explained above. In case of iHS and XP-EHH estimation we followed the previously described approach [16]. In XP-EHH analyses, we used Bantus as a reference group for all non-African populations and Europeans as a reference for the African population. Whole-genome autosomal pairwise F_ST_ values were estimated using GENEPOP 4.0 [57] and the function of pairwise F_ST_ between population *i* and the remaining populations (*d*_*i*_) was calculated in R [58].

All iHS, XP-EHH and *d*_*i*_ genome-wide results were normalized by chromosome, divided into 200-kb non-overlapping windows and binned by the number of SNPs in each window (bin size=20SNPs for XP-EHH and *d*_*i*_ analysis, and bin size=15SNPs for iHS). We further used maximum value of XP-EHH, average value of *d*_*i*_ and fraction of |iHS|>2 in each window. A two hundred kb long *TYR* window was centered in the middle of the gene and included about 41 kb of upstream and downstream sequence. Percentile rank of the *TYR* window was calculated using the genome-wide distribution of windows from the same bin.

### Phylogenetic tree reconstruction and coalescent age

We used two different approaches to reveal phylogenetic relationships among data. First, we used the median-joining (MJ) algorithm implemented in the NETWORK 4.6.1.0 software [59] to reconstruct phylogeny based on the alignment of phased sequences. The haplotype block structure of this alignment was established using HAPLOVIEW 4.2 [60]. We further reconstructed phylogenetic history and estimated time to the most recent common ancestor (TMRCA) in each haplotype block separately using local mutation rate estimates. Coalescent age was calculated using the rho statistics on the MJ network in the NETWORK 4.6.1.0 software. Mutation rate calibration was performed using 7 MYA human-chimpanzee split [61,62] assuming a 29 year generation time [63]. In addition, the age of derived non-synonymous alleles of rs1042602 (20 chromosomes in the *TYR* re-sequencing sample) and rs1126809 (10 chromosomes in the *TYR* re-sequencing sample) were estimated by two different methods. Firstly, the rho statistics was calculated using the MJ network of the complete re-sequencing alignment as described above. Secondly, a Bayesian coalescent approach was applied as implemented in BEAST 1.7.5 [64]. Data was partitioned into coding and non-coding regions. Coding nucleotides were analyzed with the SRD06 model [65]. MODELGENERATOR [66] was used to assess the substitution model of non-coding nucleotides independently in each dataset: the HKY model was chosen for sequences with the rs1042602 derived allele, and the HKY+I model was chosen for sequences with the rs1126809 derived allele. The analysis was performed using a local molecular clock of the *TYR* gene that was estimated for coding and non-coding regions separately, and using Extended Bayesian Skyline Plot as a coalescent tree prior [67]. Posterior distributions of parameters were estimated by Markov chain Monte Carlo simulation, with samples drawn every 1,000 steps over a total of 50,000,000 steps with the first 10% of samples discarded as burn-in. Three independent runs were conducted to check for convergence. Sufficient sampling of parameters was evaluated using TRACER 1.5.0 [68] and convergent runs were combined; 95% Highest Posterior Density intervals (HPD) were used as confidence intervals.

Second, we reconstructed the minimum recombination history of *TYR* haplotypes using phased Illumina genotype data for 1108 individuals. This was performed using only those *TYR* SNPs in the genotype data that were within the range of our re-sequencing alignment (n=7), or SNPs that were phylogenetically equivalent to them (n=8). Equivalency of a SNP missing from the re-sequencing project was assumed if it was in complete linkage disequilibrium (r^2^=1) with one of the re-sequenced SNPs among samples present in both re-sequencing and genotyping sample sets (Table S1). We applied filtering on our data prior to the analysis: individuals having one of the chromosomes belonging to the rare haplotype with total frequency of less than 1% in our sample were removed. In total, 942 individuals (1884 chromosomes) passed the filtering criteria. Further, ancestral recombination graphs (ARG) based on filtered and non-filtered data were reconstructed in BEAGLE and KWARG, respectively. Both programs implement a branch and bound method for determining the minimum number of recombinations in a dataset [69]. Initially, the draft ARG was reconstructed and compared to the median-joining network solution of the data. To detect fine-scale phylogeographic patterns we further genotyped eight additional sub-haplogroup defining SNPs among the individuals belonging to the particular lineage (see Table S3 for details). 

## Results

We sequenced complete coding, partial intronic and partial 5’ and 3’ flanking regions of the *TYR* gene in 81 individuals (Table S4). A total of 197 variable positions were revealed: 114 parsimony informative and 83 singletons. In total, 160 SNPs were indexed in dbSNP build 137, and 37 variants were novel. The phase of 55 singleton SNPs was successfully assigned to a particular chromosome *in vitro* by a cloning procedure. The total amount of polymorphisms in different genetic regions was as follows: 92 sites in the 5’ flanking region, 8 sites in the coding region, 96 sites in introns and 1 site in the 3’ flanking region. Five out of the eight mutations in the coding region of *TYR* were non-synonymous, of these 2 were singletons, one observed in two individuals, and 2 had minor allele frequency (MAF) higher than 0.05 in the global sample. Out of 197 markers, 137 were rare (MAF<0.05). The African sample contained the largest number of rare alleles (n=96), followed by East Asians (n=27), Europeans (n=15), South Asians (n=6), Oceanians (n=2) and Americans (n=1).

### Nucleotide diversity, mutation rate and sequence-based selection tests

**Figure 1:**
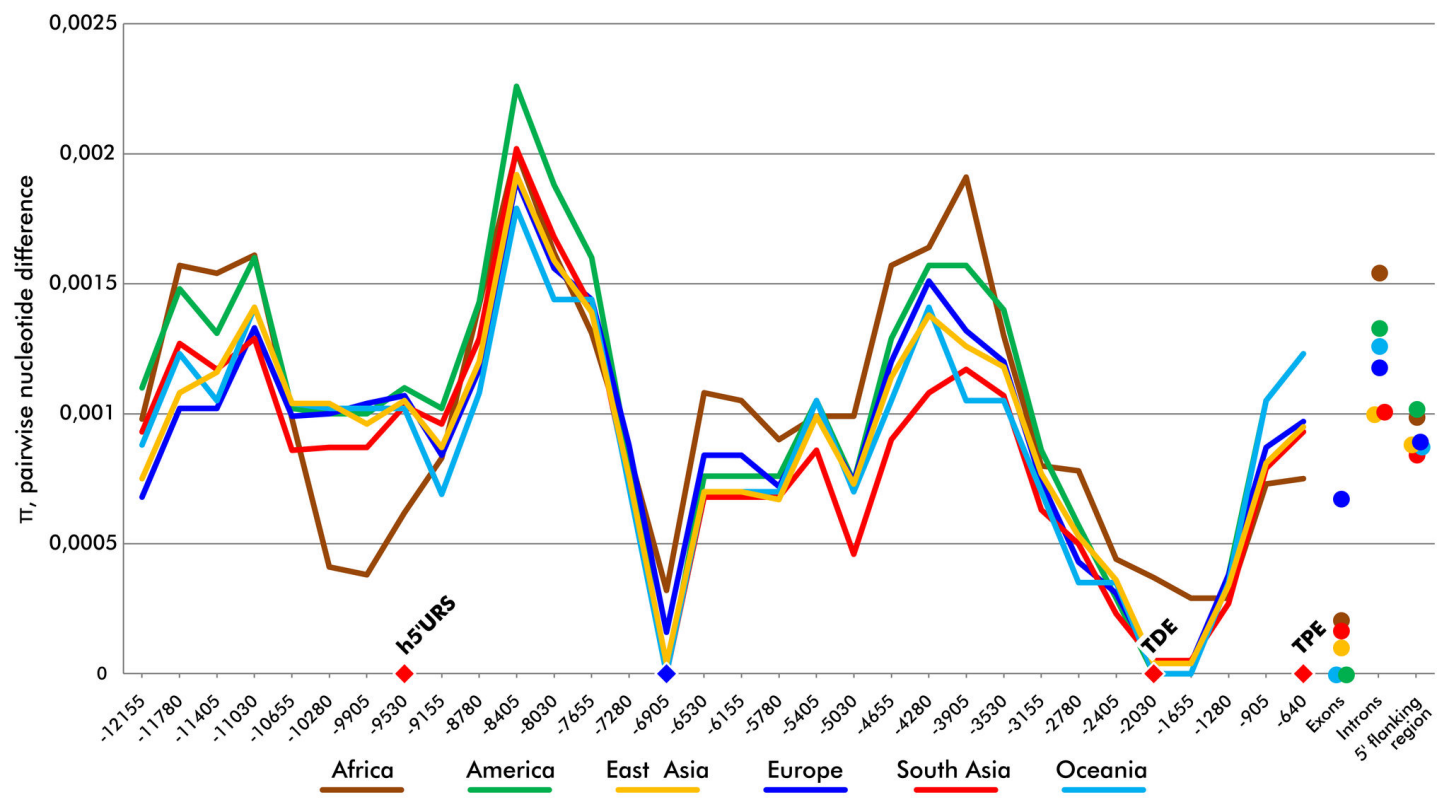
Sliding window analysis of pairwise nucleotide differences in the 5’ flanking region of the *TYR* gene. Average estimates of pairwise differences (π) at exons, introns and the complete 5’ flanking region are provided for reference. Pairwise differences in six regional population groups are shown separately. Approximate locations of previously known regulatory elements (TDE, TPE and h5’URS) are marked with red diamonds. A newly identified region with decreased genetic variability is marked with a blue diamond. Position numbers are shown relative to the first codon of the *TYR* gene. Sliding window size is 1500 bp and step size is 375 bp.

We assessed the degree of *TYR* 5’ flanking region genetic variation by sliding window nucleotide diversity (π) analysis, which estimates the average number of nucleotide differences per site between two sequences [48,70]. Additional π estimates were obtained for the combined coding, intronic and complete 5’ flanking region sequences separately (Figure 1). The average and standard deviation of nucleotide diversity among six studied populations were 0.00091±0.00007 for the 5’ flanking region, 0.00121±0.00020 for combined intronic sequence and 0.00019±0.00025 for combined coding sequence. The European sample had the highest coding π estimates among all continental groups (0.00067 in Europe, followed by 0.00021 in Africa). Nucleotide diversity in the complete re-sequencing alignment of our total human sample (0.00110) was not significantly different (p=0.30) from the average of NIEHS SNPs re-sequencing data for 647 human genes [71]. We observed two highly conserved regions with almost complete lack of diversity in our global sample, 6.9 kb and 1.8 kb upstream of the first codon position of *TYR* (Figure 1). The latter contains a previously identified conserved element, called Tyrosinase Distal Element (TDE) [30].

The Jukes-Cantor corrected average number of nucleotide substitutions per site between the human consensus sequence estimated from the data and chimpanzee (0.01447) yielded an average mutation rate at *TYR* of 3.0x10^-8^ per site per generation, assuming 7 MYA human-chimpanzee split and a human generation time of 29 years [61–63]. Mutation rate of coding and non-coding nucleotides was estimated as 8.2x10^-9^ and 3.2x10^-8^ per site per generation, respectively.

To assess the evolutionary forces acting on the human and chimpanzee branches, we calculated the ratio of the rates of non-synonymous (K_a_) and synonymous (K_s_) substitutions between the two species. The K_a_/K_s_ ratio is a measure of overall evolutionary constraints, where a value <<1 points to numerous amino acid substitutions having been eliminated by purifying selection [72]. The estimation yielded a K_a_/K_s_ ratio of 0.158, which is smaller than the genome-wide average (0.230) [73]. The difference, although not significant, comes largely from the lower rates of non-synonymous substitutions (K_a_) in our sample (0.0018 for *TYR* vs. 0.0034 genome-wide mean, p=0.94), while the rates of synonymous substitutions (K_s_) are similar (0.0114 for *TYR* vs. 0.0142 genome-wide mean, p=0.98).

Before performing neutrality tests we evaluated within population differentiation of six groups in our re-sequencing alignment using AMOVA analysis. Only the African sample showed marginally significant F_ST_ higher than zero (0.08, p=0.03, not significant after Bonferroni correction), while other populations all had slightly negative estimates pointing to the limited role of the *TYR* locus in genetic differentiation within studied (sub-) continental groups (Table S5) [74]. We further investigated our data using Tajima’s D neutrality statistics. By its original description this test assumes constant population size and lack of recombination [51]. These assumptions are overly conservative and do not normally hold in case of evolutionary history of modern humans [75]. In addition to the basic model implemented in ARLEQUIN, we used demography-corrected “best-fit” coalescent simulation approach and compared the distribution of D values obtained from neutral simulations in COSI with the empirical estimates of the *TYR* sequence data. This allowed us to include recombination into the model and to specify an explicit demographic scenario implying African origin and Out-of-Africa dispersal of anatomically modern humans [53]. To avoid possible bias which might have been introduced by the partial *TYR* re-sequencing strategy, we applied COSI simulations only to the longest consecutive sequence block in our alignment. This block included the complete 5’ flanking region and exon 1 and comprised *ca* 57% of total re-sequencing data (Figure S1 and Table S2).

The results of the neutrality tests are shown in Table 1. Only the Oceanian sample revealed significant deviation from the null hypothesis in the combined re-sequencing alignment under the basic model. In addition to Oceania, the European population similarly showed moderate departure from neutrality in the 5’ flanking region and exon 1 in the model that considers demographic history and recombination. However, European Tajima’s D value was not significant after Bonferroni correction for multiple testing. Both Europeans and Oceanians revealed positive test values pointing to an excess of intermediate frequency alleles, which could be an indication of either balancing selection or the effect of population structure. Even though the latter alternative cannot be entirely ruled out, our AMOVA analysis indicates a limited degree of within population differentiation in all studied populations (Table S5).

**Table 1:** Selection test results of six studied populations estimated using *TYR* re-sequencing alignment.

	**Africa**	**America**	**East Asia**	**Europe**	**South Asia**	**Oceania**	**Europe, w/o Israel**
**Combined alignment**							
Tajima’s D	-0.77	0.98	0.13	1.35	1.05	2.77	1.48
p-value, basic model	4.7x10^-1^	2.7x10^-1^	7.7x10^-1^	1.3x10^-1^	2.2x10^-1^	<2.0x10^-4^*	8.8x10^-2^
**5’ flanking region and exon 1**							
Tajima’s D	-0.95	1.10	0.61	1.77	1.20	2.69	1.61
p-value, basic model	3.5x10^-1^	2.1x10^-1^	4.4x10^-1^	5.2x10^-2^	1.8x10^-1^	<2.0x10^-4^*	6.6x10^-2^
p-value, “best-fit” model	5.1x10^-1^	2.1x10^-1^	5.7x10^-1^	**9.8x10^-3^**	1.6x10^-1^	<2.0x10^-4^*	**2.1x10^-2^**

Tajima’s D estimates are shown separately for the combined re-sequencing alignment (24.3 kb) and the longest consecutive sequence block, including the *TYR* 5’ flanking region and exon 1 (13.8 kb). Significance of selection statistics for the combined alignment was estimated using the basic simulation model implemented in ARLEQUIN. In addition, distribution of the statistics obtained using the COSI “best-fit” simulation model [53] was used to assess the significance of Tajima’s D values of the *TYR* 5’ flanking region and exon 1. Statistically significant test results (p<0.05) are marked in bold; results significant after Bonferroni correction for six tests (number of population groupings in our human sample) are denoted with an asterisk.

### Genotype-based selection tests

The Illumina genotyping dataset from 351 individuals was used to explore the genetic variability in the *TYR* gene in the context of genome-wide data. Data was analyzed in 200-kb non-overlapping windows. Both haplotype-based and population differentiation-based selection tests were employed. XP-EHH and iHS belong to the first class of tests and detect complete or partial selective sweeps, respectively. In the first case the selected allele is approaching or has achieved fixation, whereas in the second case selection has driven new alleles up to intermediate frequency [17,18]. The population differentiation F_ST_-based *d*_*i*_ statistic, on the other hand, is capable of detecting genomic regions with higher than expected levels of population differentiation regardless of whether selection has acted on newly arisen or preexisting variation [56]. As with other empirical data-based tests, the distinction between selection and demographic processes can be made by comparing the locus of interest, the *TYR* gene in this case, with the empirical distribution of other genes in the genome, assuming that demography affects all loci while selection affects only a subset of them. Certainly, caution is required in the interpretation because we do not actually know the proportion of the genome that has been affected by selection.

The empirical ranking order of the 200-kb window containing the *TYR* gene was determined from the genome-wide distribution of XP-EHH, iHS and *d*_*i*_ statistics calculated using the same population sample, with the 95^th^ percentile values of genome-wide distribution chosen as the significance threshold. None of the studied populations showed unusual properties in the window containing the *TYR* gene in any of the three tests performed (Table 2). The highest estimates for both haplotype-based (XP-EHH=2.11, percentile rank=0.87) and population differentiation-based selection tests (*d*_*i*_=0.93, percentile rank=0.71) were detected in the European sample. Based on these tests we cannot reject the neutral hypothesis in any of the geographic regions analyzed, but have to note that these tests have limited power to detect only certain aspects of local adaptation through positive selection.

**Table 2:** Whole-genome selection scan results of six studied populations.

	**Africa**	**America**	**East Asia**	**Europe**	**South Asia**	**Oceania**
*d*_*i*_	-1.47	-2.13	-1.70	0.93	-0.21	-0.07
*TYR* percentile rank	0.12	0.07	0.08	0.71	0.40	0.50
XP-EHH	-0.85	0.53	0.75	2.11	1.43	-0.04
*TYR* percentile rank	0.02	0.35	0.40	0.87	0.66	0.14
iHS	0.00	0.00	0.00	0.00	0.00	0.00
*TYR* percentile rank	0.49	0.62	0.55	0.54	0.54	0.58

XP-EHH, iHS and *d*_*i*_ results are shown. The percentile rank of the *TYR* 200-kb genomic window was calculated using the empirical distribution of respective statistics obtained from whole-genome data of the same population.

### Phylogenetic analysis

**Figure 2:**
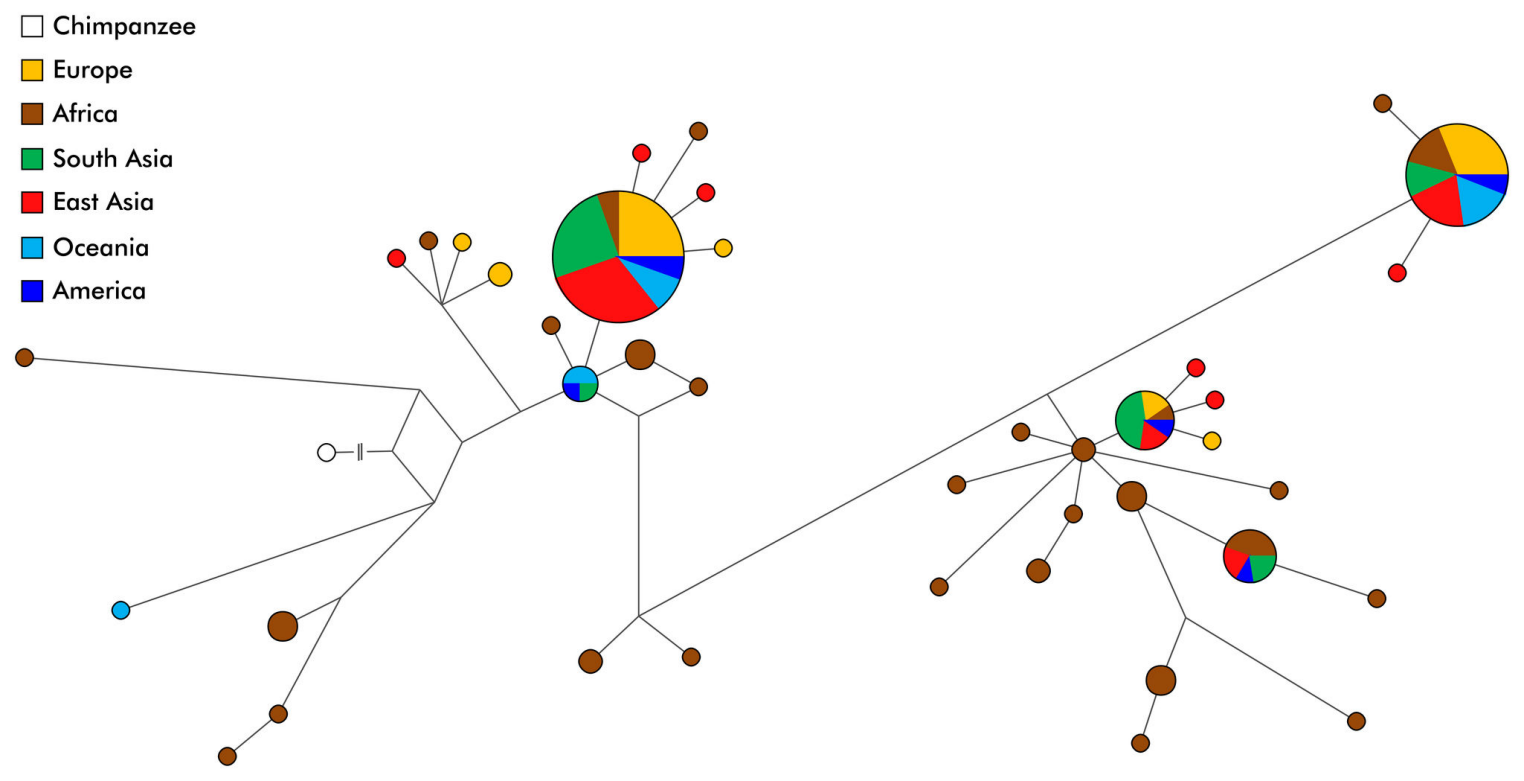
Median-joining network of *TYR* haplotypes. The network is based on the analysis of sequence data of the first haplotype block of 8634 bp length in 81 samples (162 chromosomes), color-coded by the geographic region of origin of the samples. Chimpanzee sequence (white circle) was used as an outgroup and chimpanzee specific variants have been excluded from the network output.

To further examine the geographic distribution of the allelic variation in the tyrosinase gene we reconstructed the phylogenetic tree of *TYR* haplotypes. Firstly, we determined the LD structure using HAPLOVIEW. We identified two major linkage blocks, one between 11.8 kb and 3.1 kb upstream from the first *TYR* codon and the other between 0.5 kb upstream and 106.8 kb downstream from the first *TYR* codon (Figure S2). The first block is covered by 8.6 kb uninterrupted re-sequencing data and includes 67% of our *TYR* 5’ flanking sequence. The second block spans from promoter 3’ end to exon 4 and includes a partial promoter, complete exons 1, 2, 3 and 4, plus additional intronic amplicons, and has a total length of 10.8 kb in our re-sequencing alignment. Median-joining network of the first block is presented in Figure 2. When using chimpanzee sequence as an outgroup for rooting the network, the deepest and most diverse branches appear to be among Africans, who are also represented by the largest number of individual haplotypes: 28 different haplotypes in Africa vs. 16 outside. African sequences are largely unique and share common haplotypes with non-Africans in only four cases. On the other hand, all non-Africans cluster closely together: 100 out of 114 non-African chromosomes belong to three major haplotypes or their single step relatives (Figure 2). There is no obvious geographic sub-structure among non-African variation: the majority of the branches are shared by populations with different ancestry and latitude of origin (Figure 2, Figure S3 and Figure S4). The few population specific lineages that can be observed all have low frequency. Time to the most recent common recent ancestor of the first and the second haploblock was estimated as 1.4±0.3 and 1.6±0.3 MYA, respectively. The age of the derived non-synonymous rs1042602 allele was estimated to be around 6,100±3,600 years BP by rho statistics and around 15,600 years BP (95% HPD=400–46,600 years BP) by Bayesian coalescent approach in BEAST. Time to the most recent common ancestor of samples bearing the derived non-synonymous rs1126809 allele was estimated to be around 20,400±13,600 years BP by rho statistics and around 29,400 years BP (95% HPD=1,900–68,600 years BP) by Bayesian coalescent approach.

**Figure 3:**
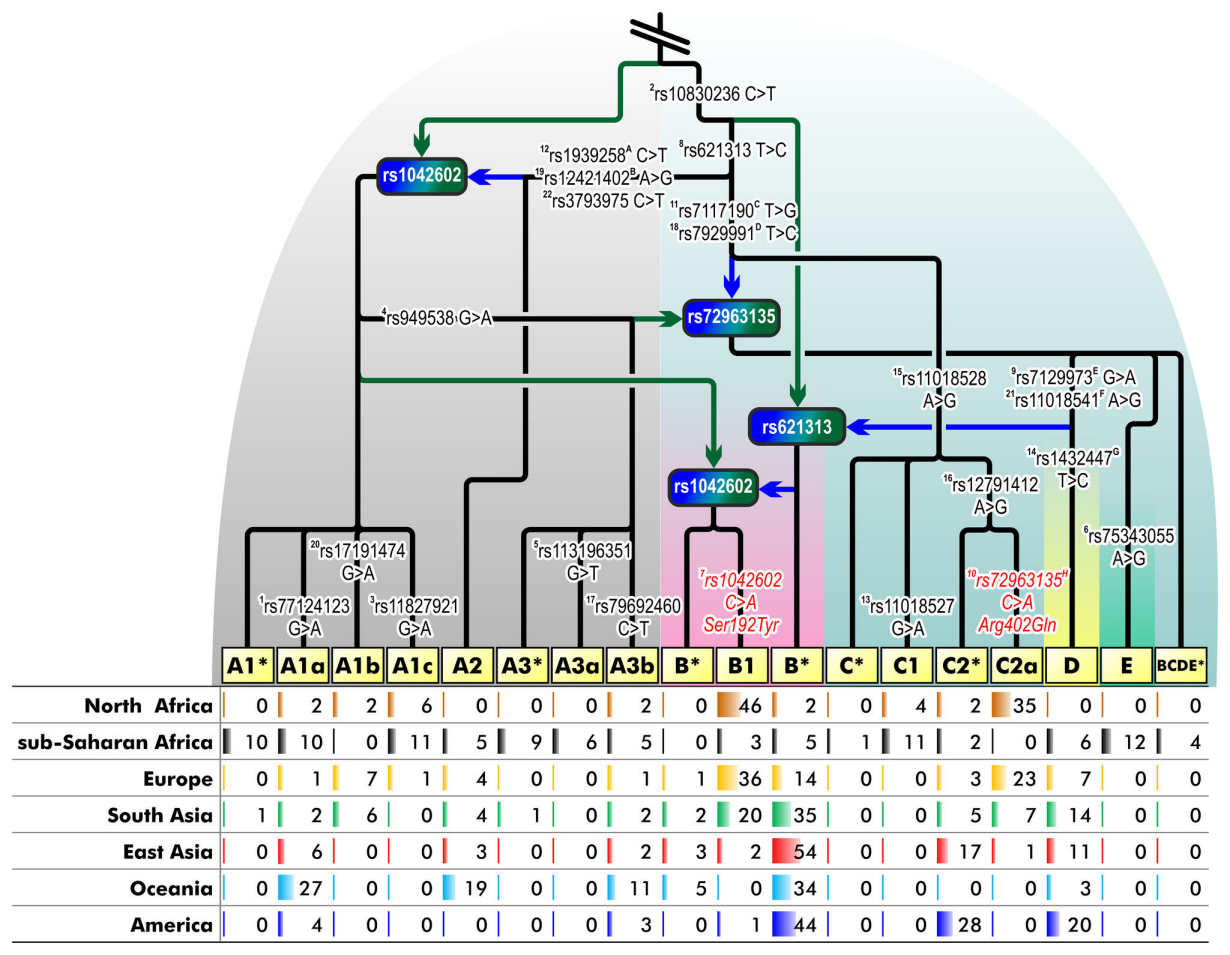
Ancestral recombination graph of *TYR* haplotypes. The tree is rooted by the chimpanzee sequence and presents recombination history of 942 worldwide samples (1884 chromosomes). Haplogroup frequency by populations is shown below the tree. Haplogroup names are shaded in yellow. Green arrows show the origin of recombination prefixes, blue arrows show the origin of recombination suffixes. Recombination points are shown by rectangles. Numerical superscript prefixes to the left of rs identifiers correspond to the relative physical position of SNPs. SNPs which were out of the range of our re-sequencing alignment are marked with superscript suffixes to the right of respective rs identifiers and correspond to the following phylogenetic equivalents in our re-sequencing data: A – rs12799137, B – rs7108676, C – rs12799347, D – rs12417632, E and F – rs5021654, G – rs7934747, H – rs1126809. Non-synonymous mutations or their phylogenetic equivalents are shown in red font with amino-acid substitutions specified. 95% confidence intervals for the detected haplogroup frequencies are given in Table S6.

The second phylogenetic analysis was performed with the Illumina genotyping sample set, 942 individuals (1884 chromosomes) were included after the removal of rare haplotypes. Eighty-one samples from the re-sequencing dataset showed complete concordance at positions examined by both Illumina genotyping and direct sequencing techniques. True minimum recombination history of the rooted tree, based on the inference of the minimum number of recombination events needed for the evolution of a given set of haplotypes, was estimated on phased data using the branch and bound algorithm in BEAGLE, and the most parsimonious outcome was visualized as an ancestral recombination graph [69]. Phylogenetic relationships of the 18 common haplotypes (haplogroups) are shown in Figure 3. In naming these major branches, we followed the nomenclature rules applied in the classification schemes of mtDNA and Y-chromosomal phylogenies for convenience [76]. The recombination graph has a deep split between clades we call A and BCDE. Haplogroup A has 8 branches found mainly in sub-Saharan Africa (56%) and Oceania (57%), whereas in other geographic regions its frequency ranges from 7 to 16%. While the Oceanian samples are represented by only three distinct lineages, the sub-Saharan African population contains all but one branches of haplogroup A. Four (A1*, A1c, A3* and A3a) out of eight A haplotypes are almost exclusively confined to sub-Saharan Africa.

Apart from Oceanian populations, the majority of non-African samples belong to the macrohaplogroup BCDE. Again, as with haplogroup A, there is limited haplotype sharing between sub-Saharan Africa and other (sub-) continental groups, with 3 out of 10 haplogroups being exclusively confined to sub-Saharan African populations. The most frequent haplogroup outside Africa is B, followed by C and D. Haplogroups B1, defined by the non-synonymous rs1042602 mutation (Ser192Tyr), and C2a, defined by rs72963135 (phylogenetically equivalent to rs1126809, Arg402Gln), are found mainly in North Africa and Europe. In contrast, the majority of Asian, Oceanian and American chromosomes belong to either B* or C2* clades. The exclusion of rare haplotypes (Figure 3 *vs.* Figure S5) did not significantly affect the geographic pattern of haplogroup distribution. For example, 13 samples with rare haplotypes bearing the non-synonymous rs1042602 mutation all originated from either North Africa or Europe. Among the 166 samples excluded in total, 60 were from sub-Saharan Africa, further highlighting the fact that this is the continental group with the highest genetic diversity (Table S1). However, despite the high overall number of distinct haplotypes in sub-Saharan Africans, the combined frequency of haplotypes with a non-synonymous mutation was among the lowest (3%) of all studied populations. 

## Discussion

In this study we have assessed the genetic variation of one of the key genes encoding melanin synthesis in six different population groups of the world. The distribution of nucleotide diversity within the 5’ flanking region is largely consistent with expectations drawn from the previously characterized location of regulatory sequence motifs. This region of the human *TYR* gene facilitates tissue-specific expression by interaction of specific transcription factors with promoter *cis*-elements including Tyrosinase Proximal Element (TPE), initiator E-box, TATA-box, SP1 site, CAAT-box, Tyrosinase Distal Element (TDE) and human 5’ upstream regulatory sequence (h5’URS) [30,77,78]. All three regulatory element blocks, TPE, TDE and h5’URS, are located inside the regions with low or average pairwise nucleotide diversity (Figure 1). There is also a significant decrease of diversity around a region 6.9 kb upstream from the first codon position of *TYR* (Figure 1). Although no regulatory motifs have been characterized in this location before, the almost complete lack of variation observed by us in all studied populations suggests the presence of a thus far undetected regulatory element.

The estimated combined *TYR* divergence from chimpanzee (0.01447) is consistent with previous local [28] and whole-genome estimates [73,79] and yields a mutation rate of 3.0x10^-8^ per site per generation. This rate is considerably higher than recent estimates of 1.2 to 1.6x10^-8^ per site per generation [80]. The difference could be partly due to local variation of mutation rates among genes and among coding and non-coding regions, and also due to our still imperfect knowledge of actual split dates in hominid evolution and/or generation times. The observed K_a_/K_s_ ratio in the *TYR* gene is lower, while not significantly different from the whole-genome average (0.158 *vs.* 0.230) [73]. Genetic variation in the human *TYR* gene coalesces around 1.4 to 1.6 MYA, which is close to the autosomal average (1.4 to 1.5 MYA) [81,82].

### Selection

There is some evidence from previous studies based on genotype data that the non-synonymous rs1042602 polymorphism (Ser192Tyr) in *TYR* exon 1 may play an important role in the evolution of light skin color in Europe and South Asia [29,33,34]. By means of a genome-wide association approach, Stokowski et al. [31] estimated this variant to explain up to 2.5% of skin reflectance difference between lightly and heavily pigmented individuals of South Asian origin. We have tested whether the *TYR* gene has been under selection in human evolution using sequence- (Tajima’s D) and genotype data-based (iHS, XP-EHH and *d*_*i*_) selection statistics. Positive Tajima’s D estimates pointed to an excess of intermediate frequency alleles in Oceania and Europe. This statistics is influenced by both population history and natural selection. For example, positive values could be yielded by balancing selection, but also by a recent bottleneck or population subdivision [83–85]. Considering the diverse geographic origin of our group of samples representing Europe, which combined various HGDP-CEPH populations from Europe and the Near East, we cannot rule out the possibility that population structure has, to some extent, affected the results of the Tajima’s D test. For example, the exclusion of five Israeli individuals lowers the European D value to 1.61 for the 5’ flanking region and *TYR* exon 1, but raises it to 1.48 for combined re-sequencing alignment without substantially affecting the probability estimates (Table 1). The presented AMOVA analysis of *TYR* re-sequencing data similarly did not detect any significant differentiation within population in either the European or the Oceanian sample, indicating a limited effect of population structure on the reported Tajima’s D values (Table S5). Furthermore, populations from the Near East and Europe have previously been shown to share signals of selection in other pigmentation genes, including *SLC24A5* and *KITLG* [16], and the majority of the individual samples used here have previously been examined by STRUCTURE-like analyses and clustered into similar (sub-) continental groups as defined by us in the present study (references provided in Table S1). We conclude that although a minor effect of population structure is possible, it seems unlikely to be the major cause of the significantly positive Tajima’s D in our European sample.

While modeling population history of the Oceanian populations we used as a proxy the “best-fit” demographic model of East Asians. The interpretation of the results of significantly positive Tajima’s D in our study should thus be considered with caution as the demographic parameters of population history of Papuans and Bougainville Islanders, who formed the Oceanian group, are likely to be quite different from East Asians, considering their isolation and existing genetic data on these populations [86–88]. It is possible that small founding population size, long-term isolation and high level of genetic drift, rather than natural selection, are responsible for the observed pattern of genetic variation in the *TYR* locus in Oceania.

Other selection scanning approaches employed here – extended haplotype homozygosity-based iHS and XP-EHH tests, and population differentiation-based *d*_*i*_ tests – did not reject the neutral hypothesis in the *TYR* gene and adjacent genomic regions in any of the population groups considered in this study. Nevertheless, even if significantly positive Tajima’s D values do indeed indicate traces of balancing selection and/or population structure in the Oceanian and European samples, the lack of a selection signal in our genotyping data is not unexpected: Tajima’s D utilizes different evolutionary assumptions and operates on a different time scale. For example, the haplotype homozygosity tests are only capable of detecting a particular kind of selection, one involving a recent selective sweep, and, thus, more ancient selection events and those involving standing variation or multiple low effect size mutations may go undetected [85,89].

### Geographic distribution of the functional variation of *TYR*

The ancestral recombination graph based on the genotyping results of 942 individuals (Figure 3) shows that haplogroup B is frequent and widely spread among non-African populations. Europeans harbor the highest frequency of one of its branches, B1, which is defined by a non-synonymous rs1042602 polymorphism. B1 and B* (containing all other branches of B except for B1) show contrasting frequency patterns: while B* is common in East Asia, Oceania and America, B1 is virtually absent there. It is interesting to note that B1 is also rare in sub-Saharan Africans (0.03, 95%CI=0.01–0.08; 95% confidence intervals for detected haplogroup frequencies are given in Table S6). In contrast, B1 chromosomes are frequent in North Africa among Algerian Mozabites, where they occur at 46% frequency (95%CI=0.34–0.59). The same is true for the other non-synonymous mutation, rs1126809. Its phylogenetic equivalent in our genotyping panel, rs72963135 (r^2^=1.0), is absent in darkly pigmented sub-Saharan populations, while it reaches 35% frequency in North African Mozabites. Moreover, non-synonymous variation in sub-Saharans is represented only by a single private mutation in the re-sequenced Kenyans (Table S4). The highest combined frequency of non-synonymous derived alleles of the *TYR* gene was observed in Europe (0.59, 95%CI=0.55–0.64) and North Africa (0.81, 95%CI=0.69–0.90). The majority of this variation is due to the presence of either rs1042602 in exon 1 or rs1126809 in exon 4 tagged by the rs72963135 polymorphism in the genotyping data. The latter mutation defines haplogroup C2a, which is again represented mainly in European (0.23, 95%CI=0.20–0.27) and North African samples (0.35, 95%CI=0.24–0.49). The human ancestral alleles of both rs1042602 (Ser192Tyr) and rs1126809 (Arg402Gln) are almost completely conserved among different species, and mutations at these positions are classified as probably having damaging impact on protein function by the PolyPhen-2 tool [90]. Functional assays of Tyr192 and Gln402 alleles have also revealed about 40% and up to 75% reduced enzymatic activity, respectively, confirming the *in silico* results [91,92]. In total, 72% of the chromosomes in our genotyping dataset that were inferred to carry at least one non-synonymous difference compared to the ancestral human sequence were confined to European and North African samples. The sharing of this pattern between these two populations is not surprising considering that both are characterized by low pigmentation level. Furthermore, the present-day North Africans have been proposed to derive from several distinctive migrations, including an ancient back-migration from Europe and the Near East [93]. The proportion of the ancestry component shared with Europeans in the HGDP-CEPH Mozabite sample has been estimated to be around 80% [42,43,94] and, therefore, the allele sharing patterns observed in the *TYR* gene are likely due to common demographic history rather than convergent adaptation.

Similarly to the results observed here for *TYR*, Harding et al. [15] also detected the absence of non-synonymous alleles in the *MC1R* gene among sub-Saharan Africans and an excess of non-synonymous mutations in Europe. This locus, coding for the melanocortin 1 receptor, is responsible for the activation of the dark eumelanin biosynthesis pathway [95]. Mutations in *MC1R*, which lead to higher pheomelanin level, lower photoprotection and increased vitamin D production, could have been advantageous when humans dispersed from Africa to environments with lower annual UV levels. However, no traces of selective enhancement have been detected, suggesting that the relaxation of a strong functional constraint in low UV environment may have been an important factor in the evolution of the *MC1R* gene [15]. Thus, the moderate excess of intermediate frequency polymorphisms in *TYR*, the highest incidence of non-synonymous mutations and failure to detect any directional positive selection by haplotype homozygosity tests can be ascribed to the relaxation of selective pressure in the European subcontinent. This is further confirmed by the highest coding nucleotide diversity (0.00067) observed in our European sample. A similar pattern was found in the *MC1R* gene [15,96] and is quite unusual on the genome-wide level, as more generally populations with African ancestry have the highest nucleotide diversity [97]. Furthermore, studies on these two loci are consistent with the view that accumulation of functional variation in pigmentation genes is restricted in high UV environment. In fact, in our data both sub-Saharan African and Oceanian samples almost completely lack non-synonymous *TYR* polymorphisms and show a virtual fixation of the amino-acid sequence ancestral to anatomically modern humans. However, it is intriguing to observe that South Asians possess considerable variation in the *TYR* gene. We speculate that this could be explained by the mosaic pattern of their geographic ancestry, as our sample includes populations from both the northwestern and southern parts of the Indian peninsula (Table S1).

Although we did not detect any traces of selective sweeps in *TYR* using haplotype-based iHS and XP-EHH tests, there is evidence from previous studies for strong sweeps acting upon the *SLC24A5* and *SLC45A2* genes in Europe [16,17,98]. Therefore, it is possible that various evolutionary mechanisms have affected genetic diversity within different pigmentation loci: relaxation of functional constraints or weak balancing selection may have been responsible for the excess of European-specific non-synonymous alleles in *MC1R*, and, as we show here, in *TYR*, while strong positive selection has led to the nearly complete fixation of amino-acid changing substitutions in the *SLC24A5* and *SLC45A2* genes.

Variation patterns in different pigmentation genes suggest that skin lightening in Eurasia was a gradual multistage process. It included the evolution of the *KITLG* gene in the proto-Eurasian population and convergent evolution of other loci after the split of proto-Europeans and proto-East Asians. This is reflected by the European-specific sweep in the *SLC24A5, SLC45A2* and *TYRP1* genes which have been dated to 19,000 to 11,000 years BP [23,99]. The tyrosinase haplogroups B1 and C2a are defined by non-synonymous substitutions and are common in Europe and North Africa. Haplogroup B1 coalesces at 15,600 and 6,100, and haplogroup C2a at 29,400 and 20,400 years BP as calculated by the Bayesian-based and rho statistics, respectively. These dates suggest that both non-synonymous mutations occurred after the split of the East and West Eurasian populations. However, the TMRCA of 6,100±3,600 years of haplogroup B1 estimated by the rho approach is still surprisingly young, considering its more than 20% frequency in Europe, North Africa and South Asia. The point estimates of these coalescent times should be interpreted with caution because of our still imperfect understanding of the mutation rates at different genetic loci. It should be noted here that our local calibration of the *TYR* gene mutation rate (3.0x10^-8^ per site per generation) is almost three times higher than the genome average rate (1.2x10^-8^ per site per generation) [80]. Applying the slower genome-wide rate would have yielded a 17 MYA coalescent date of the TMRCA of the human and chimpanzee *TYR* genes, which would have been in conflict with paleontological evidence. Different computational methods may also return non-identical results. The more than two-fold difference in mean coalescence times of B1 as calculated by MJ network-based rho (6,100 years BP) and Bayesian coalescence-based BEAST (15,600 years BP) approaches can be ascribed to the differences in parameter settings of the two TMRCA evaluation techniques. Overall, the most recent common ancestors of both haplogroups appear to, at least partly, overlap with the time period at which the selective sweeps at three other pigmentation genes, *SLC24A5, SLC45A2* and *TYRP1*, have been estimated [23,99]. It is therefore possible that *TYR* polymorphisms have also contributed to the same adaptive process in the genetic ancestry of the West Eurasian population. This process may have been driven by various factors, including climatic, cultural and demographic changes during the cooler LGM and early post-LGM period: decrease in the winter UVR level and extensive use of protective clothing, both affecting vitamin D bioavailability, and population size growth [3,23,100]. Our results also provide evidence that besides the non-synonymous polymorphisms, variation in the 5’ flanking sequence of the *TYR* gene has potentially played an important role in the adaptation to UV environment.

## Supporting Information

Figure S1 **Graphical representation of *TYR* regions sequenced in the course of the current study.** The locations of flanking regions (pink), introns (dotted line) and exons (yellow) are shown. Regions sequenced are shaded in blue.
(TIF)

Figure S2 **HAPLOVIEW GOLD heatmap of SNPs polymorphic in the complete re-sequencing alignment.** Rare SNPs with minor allele frequency less than 0.05 are not shown. D’ values under 1 are indicated. The color scheme from blue to red indicates increasing D’ values.
(TIF)

Figure S3 **Median-joining network of the second haplotype block identified in the course of the *TYR* re-sequencing project.** 162 haplotypes are shown; only polymorphisms belonging to the second haplotype block (see Figure S2) are included. Nodes are proportional to haplotype frequency; branch lengths are proportional to the number of mutations. Geographic distribution of each haplotype is coded by color according to the legend. The network is rooted by chimpanzee sequence (white); only human-specific polymorphisms are shown.
(TIF)

Figure S4 **Median-joining network of the complete re-sequencing alignment.** 162 haplotypes are shown; polymorphisms belonging to the *TYR* gene and its 5’ and 3’ flanking sequences are included. Nodes are proportional to haplotype frequency; branch lengths are proportional to the number of mutations. Geographic distribution of each haplotype is coded by color according to the legend. The network is rooted by chimpanzee sequence (white); only human-specific polymorphisms are shown. Callouts show the positions of two non-synonymous polymorphisms and their phylogenetic equivalents used in the genotyping project.
(TIF)

Figure S5 **Ancestral recombination graph showing phylogenetic relationships between 1108 samples used in the present study.** The complete dataset, including samples with rare haplotypes, is shown. ARG was reconstructed using the KWARG software and represents one of the possible phylogenetic solutions of non-filtered *TYR* genotyping data. Haplogroup frequency by populations is shown below the tree. “P” and “S” indicate the origin of recombination prefix and suffix, respectively. Recombination points are shown in blue ovals. Individual haplotypes are shown in red ovals and coded according to Table S1.
(PDF)

Table S1 **List of samples used in the re-sequencing project, whole-genome selection scan and ancestral recombination graph reconstruction.** “Illumina TYR haplotype 1” and “Illumina TYR haplotype 2” denote the phased haplotypes of each sample estimated from *TYR* SNPs. “Rare haplotype” means that one of the chromosomes belongs to a haplotype with a total frequency of less than 1% in our human sample. “Haplotype code” corresponds to individual haplotypes in Figure S5.
(XLSX)

Table S2 **Detailed information of *TYR* regions sequenced in the course of the current study.** Genomic positions are given according to hg19, GRCh37 human reference sequence.
(XLSX)

Table S3 **Additional markers genotyped for the ARG reconstruction.** Haplogroup defining motifs from the Illumina genotyping array are shown. Additional markers were typed only among samples belonging to the particular haplogroup.
(XLSX)

Table S4 **Single nucleotide polymorphisms detected in the course of the re-sequencing project.** Genomic positions are given according to hg19, GRCh37 human reference sequence and rs identifiers according to dbSNP 137. “S” corresponds to a singleton, “S*” corresponds to a singleton the phase of which was determined by cloning.
(XLSX)

Table S5 **Within population differentiation F_ST_ values estimated by AMOVA analysis.** Negative F_ST_ values suggest that the true F_ST_ measures are probably not significantly different from 0 and indicate a limited role of the *TYR* gene in the genetic differentiation within studied (sub-) continental groups.
(XLSX)

Table S6 **Frequency and 95% confidence intervals of 18 haplogroups detected from the Illumina genotyping dataset.** Haplogroup frequencies and 95% confidence intervals within the studied (sub-) continental groups are shown.
(XLSX)

## References

[1] Relethford JH (2002) Apportionment of global human genetic diversity based on craniometrics and skin color. Am J Phys Anthropol 118: 393-398. doi:10.1002/ajpa.10079. PubMed: 12124919. 12124919 10.1002/ajpa.10079

[2] Jablonski NG, Chaplin G (2000) The evolution of human skin coloration. J Hum Evol 39: 57-106. doi:10.1006/jhev.2000.0403. PubMed: 10896812. 10896812 10.1006/jhev.2000.0403

[3] Jablonski NG (2004) The evolution of human skin and skin color. Annu Rev Anthropol 33: 585-623. doi:10.1146/annurev.anthro.33.070203.143955.

[4] Loomis WF (1967) Skin-pigment regulation of vitamin-D biosynthesis in man. Science 157: 501-506. doi:10.1126/science.157.3788.501. PubMed: 6028915. 6028915 10.1126/science.157.3788.501

[5] Branda RF, Eaton JW (1978) Skin color and nutrient photolysis: an evolutionary hypothesis. Science 201: 625-626. doi:10.1126/science.675247. PubMed: 675247. 675247 10.1126/science.675247

[6] Aoki K (2002) Sexual selection as a cause of human skin colour variation: Darwin’s hypothesis revisited. Ann Hum Biol 29: 589-608. doi:10.1080/0301446021000019144. PubMed: 12573076. 12573076 10.1080/0301446021000019144

[7] Elias PM, Menon G, Wetzel BK, Williams JW (2010) Barrier Requirements as the Evolutionary "Driver" of Epidermal Pigmentation in Humans. Am J Hum Biol 22: 526-537. doi:10.1002/ajhb.21043. PubMed: 20209486. 20209486 10.1002/ajhb.21043 PMC3071612

[8] Parra EJ (2007) Human Pigmentation Variation: Evolution, Genetic Basis, and Implications for Public Health. Yearbook Phys Anthropol, Vol 50 50: 85-105. PubMed: 18046745. 10.1002/ajpa.20727 18046745

[9] Montoliu L, Oetting WS, Bennett DC (2012) Color Genes. European Society for Pigment Cell Research.

[10] Anno S, Ohshima K, Abe T (2010) Approaches to understanding adaptations of skin color variation by detecting gene-environment interactions. Expert Rev Mol Diagn 10: 987-991. doi:10.1586/erm.10.90. PubMed: 21080816. 21080816 10.1586/erm.10.90

[11] Sturm RA, Duffy DL (2012) Human pigmentation genes under environmental selection. Genome Biol 13: 248. doi:10.1186/gb-2012-13-9-248. PubMed: 23110848. 23110848 10.1186/gb-2012-13-9-248 PMC3491390

[12] Lamason RL, Mohideen MA, Mest JR, Wong AC, Norton HL et al. (2005) SLC24A5, a putative cation exchanger, affects pigmentation in zebrafish and humans. Science 310: 1782-1786. 16357253 10.1126/science.1116238

[13] Kenny EE, Timpson NJ, Sikora M, Yee M-C, Moreno-Estrada A et al. (2012) Melanesian Blond Hair Is Caused by an Amino Acid Change in TYRP1. Science 336: 554. doi:10.1126/science.1217849. PubMed: 22556244. 22556244 10.1126/science.1217849 PMC3481182

[14] Lalueza-Fox C, Römpler H, Caramelli D, Stäubert C, Catalano G et al. (2007) A Melanocortin 1 Receptor Allele Suggests Varying Pigmentation Among Neanderthals. Science 318: 1453-1455. doi:10.1126/science.1147417. PubMed: 17962522. 17962522 10.1126/science.1147417

[15] Harding RM, Healy E, Ray AJ, Ellis NS, Flanagan N et al. (2000) Evidence for variable selective pressures at MC1R. Am J Hum Genet 66: 1351-1361. doi:10.1086/302863. PubMed: 10733465. 10733465 10.1086/302863 PMC1288200

[16] Pickrell JK, Coop G, Novembre J, Kudaravalli S, Li JZ et al. (2009) Signals of recent positive selection in a worldwide sample of human populations. Genome Res 19: 826-837. doi:10.1101/gr.087577.108. PubMed: 19307593. 19307593 10.1101/gr.087577.108 PMC2675971

[17] Sabeti PC, Varilly P, Fry B, Lohmueller J, Hostetter E et al. (2007) Genome-wide detection and characterization of positive selection in human populations. Nature 449: 913-918. doi:10.1038/nature06250. PubMed: 17943131. 17943131 10.1038/nature06250 PMC2687721

[18] Voight BF, Kudaravalli S, Wen X, Pritchard JK (2006) A map of recent positive selection in the human genome. PLOS Biol 4: e72. doi:10.1371/journal.pbio.0040072. PubMed: 16494531. 16494531 10.1371/journal.pbio.0040072 PMC1382018

[19] Williamson SH, Hubisz MJ, Clark AG, Payseur BA, Bustamante CD et al. (2007) Localizing recent adaptive evolution in the human genome. PLOS Genet 3: 901-915. PubMed: 17542651. 10.1371/journal.pgen.0030090 PMC1885279 17542651

[20] Rana BK, Hewett-Emmett D, Jin L, Chang BH, Sambuughin N et al. (1999) High polymorphism at the human melanocortin 1 receptor locus. Genetics 151: 1547-1557. PubMed: 10101176. 10101176 10.1093/genetics/151.4.1547 PMC1460552

[21] McEvoy B, Beleza S, Shriver MD (2006) The genetic architecture of normal variation in human pigmentation: an evolutionary perspective and model. Hum Mol Genet 15: R176–81. PubMed: 16987881. 16987881 10.1093/hmg/ddl217

[22] Meyer M, Kircher M, Gansauge MT, Li H, Racimo F et al. (2012) A high-coverage genome sequence from an archaic Denisovan individual. Science 338: 222-226. doi:10.1126/science.1224344. PubMed: 22936568. 22936568 10.1126/science.1224344 PMC3617501

[23] Beleza S, Dos Santos AM, McEvoy B, Alves I, Martinho C et al. (2012) The timing of pigmentation lightening in Europeans. Mol Biol Evol, 30: 24–35. PubMed: 22923467. 22923467 10.1093/molbev/mss207 PMC3525146

[24] Miller CT, Beleza S, Pollen AA, Schluter D, Kittles RA et al. (2007) cis-Regulatory changes in Kit ligand expression and parallel evolution of pigmentation in sticklebacks and humans. Cell 131: 1179-1189. doi:10.1016/j.cell.2007.10.055. PubMed: 18083106. 18083106 10.1016/j.cell.2007.10.055 PMC2900316

[25] Lao O, de Gruijter JM, van Duijn K, Navarro A, Kayser M (2007) Signatures of positive selection in genes associated with human skin pigmentation as revealed from analyses of single nucleotide polymorphisms. Ann Hum Genet 71: 354-369. doi:10.1111/j.1469-1809.2006.00341.x. PubMed: 17233754. 17233754 10.1111/j.1469-1809.2006.00341.x

[26] Edwards M, Bigham A, Tan J, Li S, Gozdzik A et al. (2010) Association of the OCA2 polymorphism His615Arg with melanin content in east Asian populations: further evidence of convergent evolution of skin pigmentation. PLOS Genet 6: e1000867 PubMed: 20221248. 20221248 10.1371/journal.pgen.1000867 PMC2832666

[27] Myles S, Somel M, Tang K, Kelso J, Stoneking M (2007) Identifying genes underlying skin pigmentation differences among human populations. Hum Genet 120: 613-621. PubMed: 16977434. 16977434 10.1007/s00439-006-0256-4

[28] Alonso S, Izagirre N, Smith-Zubiaga I, Gardeazabal J, Díaz-Ramón JL et al. (2008) Complex signatures of selection for the melanogenic loci TYR, TYRP1 and DCT in humans. BMC Evol Biol 8: 74. doi:10.1186/1471-2148-8-74. PubMed: 18312627. 18312627 10.1186/1471-2148-8-74 PMC2292700

[29] Norton HL, Kittles RA, Parra E, McKeigue P, Mao X et al. (2007) Genetic evidence for the convergent evolution of light skin in Europeans and East Asians. Mol Biol Evol 24: 710-722. PubMed: 17182896. 17182896 10.1093/molbev/msl203

[30] Ray K, Chaki M, Sengupta M (2007) Tyrosinase and ocular diseases: Some novel thoughts on the molecular basis of oculocutaneous albinism type 1. Prog Retin Eye Res 26: 323-358. doi:10.1016/j.preteyeres.2007.01.001. PubMed: 17355913. 17355913 10.1016/j.preteyeres.2007.01.001

[31] Stokowski RP, Pant PV, Dadd T, Fereday A, Hinds DA et al. (2007) A genomewide association study of skin pigmentation in a South Asian population. Am J Hum Genet 81: 1119-1132. doi:10.1086/522235. PubMed: 17999355. 17999355 10.1086/522235 PMC2276347

[32] Sulem P, Gudbjartsson DF, Stacey SN, Helgason A, Rafnar T et al. (2007) Genetic determinants of hair, eye and skin pigmentation in Europeans. Nat Genet 39: 1443-1452. doi:10.1038/ng.2007.13. PubMed: 17952075. 17952075 10.1038/ng.2007.13

[33] Shriver MD, Parra EJ, Dios S, Bonilla C, Norton H et al. (2003) Skin pigmentation, biogeographical ancestry and admixture mapping. Hum Genet 112: 387-399. PubMed: 12579416. 12579416 10.1007/s00439-002-0896-y

[34] Beleza S, Johnson NA, Candille SI, Absher DM, Coram MA et al. (2013) Genetic architecture of skin and eye color in an African–European admixed population. PLOS Genet 9: e1003372. 23555287 10.1371/journal.pgen.1003372 PMC3605137

[35] Gudbjartsson DF, Sulem P, Stacey SN, Goldstein AM, Rafnar T et al. (2008) ASIP and TYR pigmentation variants associate with cutaneous melanoma and basal cell carcinoma. Nat Genet 40: 886-891. doi:10.1038/ng.161. PubMed: 18488027. 18488027 10.1038/ng.161

[36] Nan H, Kraft P, Hunter DJ, Han J (2009) Genetic variants in pigmentation genes, pigmentary phenotypes, and risk of skin cancer in Caucasians. Int J Cancer 125: 909-917. doi:10.1002/ijc.24327. PubMed: 19384953. 19384953 10.1002/ijc.24327 PMC2700213

[37] Ibarrola-Villava M, Hu HH, Guedj M, Fernandez LP, Descamps V et al. (2012) MC1R, SLC45A2 and TYR genetic variants involved in melanoma susceptibility in southern European populations: results from a meta-analysis. Eur J Cancer 48: 2183-2191. doi:10.1016/j.ejca.2012.03.006. PubMed: 22464347. 22464347 10.1016/j.ejca.2012.03.006

[38] Izagirre N, García I, Junquera C, de la Rúa C, Alonso S (2006) A scan for signatures of positive selection in candidate loci for skin pigmentation in humans. Mol Biol Evol 23: 1697-1706. doi:10.1093/molbev/msl030. PubMed: 16757656. 16757656 10.1093/molbev/msl030

[39] Candille SI, Absher DM, Beleza S, Bauchet M, McEvoy B et al. (2012) Genome-wide association studies of quantitatively measured skin, hair, and eye pigmentation in four European populations. PLOS ONE 7: e48294. doi:10.1371/journal.pone.0048294. PubMed: 23118974. 23118974 10.1371/journal.pone.0048294 PMC3485197

[40] de Gruijter JM, Lao O, Vermeulen M, Xue Y, Woodwark C et al. (2011) Contrasting signals of positive selection in genes involved in human skin-color variation from tests based on SNP scans and resequencing. Investig Genet 2: 24. doi:10.1186/2041-2223-2-24. PubMed: 22133426. 10.1186/2041-2223-2-24 PMC3287149 22133426

[41] Cann HM, de Toma C, Cazes L, Legrand MF, Morel V et al. (2002) A human genome diversity cell line panel. Science 296: 261-262. doi:10.1126/science.296.5566.261a. PubMed: 11954565. 11954565 10.1126/science.296.5566.261b

[42] Li JZ, Absher DM, Tang H, Southwick AM, Casto AM et al. (2008) Worldwide human relationships inferred from genome-wide patterns of variation. Science 319: 1100-1104. doi:10.1126/science.1153717. PubMed: 18292342. 18292342 10.1126/science.1153717

[43] Behar DM, Yunusbayev B, Metspalu M, Metspalu E, Rosset S et al. (2010) The genome-wide structure of the Jewish people. Nature 466: 238-242. doi:10.1038/nature09103. PubMed: 20531471. 20531471 10.1038/nature09103

[44] Chaubey G, Metspalu M, Choi Y, Mägi R, Romero IG et al. (2011) Population genetic structure in Indian Austroasiatic speakers: the role of landscape barriers and sex-specific admixture. Mol Biol Evol 28: 1013-1024. doi:10.1093/molbev/msq288. PubMed: 20978040. 20978040 10.1093/molbev/msq288 PMC3355372

[45] Metspalu M, Romero IG, Yunusbayev B, Chaubey G, Mallick CB et al. (2011) Shared and unique components of human population structure and genome-wide signals of positive selection in South Asia. Am J Hum Genet 89: 731-744. doi:10.1016/j.ajhg.2011.11.010. PubMed: 22152676. 22152676 10.1016/j.ajhg.2011.11.010 PMC3234374

[46] Stephens M, Donnelly P (2003) A comparison of bayesian methods for haplotype reconstruction from population genotype data. Am J Hum Genet 73: 1162-1169. doi:10.1086/379378. PubMed: 14574645. 14574645 10.1086/379378 PMC1180495

[47] Stephens M, Smith NJ, Donnelly P (2001) A new statistical method for haplotype reconstruction from population data. Am J Hum Genet 68: 978-989. doi:10.1086/319501. PubMed: 11254454. 11254454 10.1086/319501 PMC1275651

[48] Librado P, Rozas J (2009) DnaSP v5: a software for comprehensive analysis of DNA polymorphism data. Bioinformatics 25: 1451-1452. doi:10.1093/bioinformatics/btp187. PubMed: 19346325. 19346325 10.1093/bioinformatics/btp187

[49] Purcell S, Neale B, Todd-Brown K, Thomas L, Ferreira MA et al. (2007) PLINK: a tool set for whole-genome association and population-based linkage analyses. Am J Hum Genet 81: 559-575. doi:10.1086/519795. PubMed: 17701901. 17701901 10.1086/519795 PMC1950838

[50] Browning SR, Browning BL (2007) Rapid and accurate haplotype phasing and missing-data inference for whole-genome association studies by use of localized haplotype clustering. Am J Hum Genet 81: 1084-1097. doi:10.1086/521987. PubMed: 17924348. 17924348 10.1086/521987 PMC2265661

[51] Tajima F (1989) Statistical Method for Testing the Neutral Mutation Hypothesis by DNA Polymorphism. Genetics 123: 585-595. PubMed: 2513255. 2513255 10.1093/genetics/123.3.585 PMC1203831

[52] Excoffier L, Lischer HE (2010) Arlequin suite ver 3.5: a new series of programs to perform population genetics analyses under Linux and Windows. Mol Ecol Resour 10: 564-567. doi:10.1111/j.1755-0998.2010.02847.x. PubMed: 21565059. 21565059 10.1111/j.1755-0998.2010.02847.x

[53] Schaffner SF, Foo C, Gabriel S, Reich D, Daly MJ et al. (2005) Calibrating a coalescent simulation of human genome sequence variation. Genome Res 15: 1576-1583. doi:10.1101/gr.3709305. PubMed: 16251467. 16251467 10.1101/gr.3709305 PMC1310645

[54] Ferrer-Admetlla A, Sikora M, Laayouni H, Esteve A, Roubinet F et al. (2009) A natural history of FUT2 polymorphism in humans. Mol Biol Evol 26: 1993-2003. doi:10.1093/molbev/msp108. PubMed: 19487333. 19487333 10.1093/molbev/msp108

[55] the International HapMap Consortium (2003) The International HapMap Project. Nature 426: 789-796. doi:10.1038/nature02168. PubMed: 14685227. 14685227 10.1038/nature02168

[56] Akey JM, Ruhe AL, Akey DT, Wong AK, Connelly CF et al. (2010) Tracking footprints of artificial selection in the dog genome. Proc Natl Acad Sci U S A 107: 1160-1165. doi:10.1073/pnas.0909918107. PubMed: 20080661. 20080661 10.1073/pnas.0909918107 PMC2824266

[57] Rousset F (2008) genepop’007: a complete re-implementation of the genepop software for Windows and Linux. Mol Ecol Resour 8: 103-106. doi:10.1111/j.1471-8286.2007.01931.x. PubMed: 21585727. 21585727 10.1111/j.1471-8286.2007.01931.x

[58] Core R Team (2013) R: A Language and Environment for Statistical. Computing.

[59] Bandelt HJ, Forster P, Röhl A (1999) Median-joining networks for inferring intraspecific phylogenies. Mol Biol Evol 16: 37-48. doi:10.1093/oxfordjournals.molbev.a026036. PubMed: 10331250. 10331250 10.1093/oxfordjournals.molbev.a026036

[60] Barrett JC, Fry B, Maller J, Daly MJ (2005) Haploview: analysis and visualization of LD and haplotype maps. Bioinformatics 21: 263-265. doi:10.1093/bioinformatics/bth457. PubMed: 15297300. 15297300 10.1093/bioinformatics/bth457

[61] Langergraber KE, Prüfer K, Rowney C, Boesch C, Crockford C et al. (2012) Generation times in wild chimpanzees and gorillas suggest earlier divergence times in great ape and human evolution. Proc Natl Acad Sci U.S.A. PubMed: 22891323 10.1073/pnas.1211740109 PMC3465451 22891323

[62] Scally A, Dutheil JY, Hillier LW, Jordan GE, Goodhead I et al. (2012) Insights into hominid evolution from the gorilla genome sequence. Nature 483: 169-175. doi:10.1038/nature10842. PubMed: 22398555. 22398555 10.1038/nature10842 PMC3303130

[63] Fenner JN (2005) Cross-cultural estimation of the human generation interval for use in genetics-based population divergence studies. Am J Phys Anthropol 128: 415-423. doi:10.1002/ajpa.20188. PubMed: 15795887. 15795887 10.1002/ajpa.20188

[64] Drummond AJ, Suchard MA, Xie D, Rambaut A (2012) Bayesian phylogenetics with BEAUti and the BEAST 1.7. Mol Biol Evol 29: 1969-1973. 22367748 10.1093/molbev/mss075 PMC3408070

[65] Shapiro B, Rambaut A, Drummond AJ (2006) Choosing appropriate substitution models for the phylogenetic analysis of protein-coding sequences. Mol Biol Evol 23: 7-9. PubMed: 16177232. 16177232 10.1093/molbev/msj021

[66] Keane TM, Creevey CJ, Pentony MM, Naughton TJ, McLnerney JO (2006) Assessment of methods for amino acid matrix selection and their use on empirical data shows that ad hoc assumptions for choice of matrix are not justified. BMC Evol Biol 6: 29. doi:10.1186/1471-2148-6-29. PubMed: 16563161. 16563161 10.1186/1471-2148-6-29 PMC1435933

[67] Heled J, Drummond AJ (2008) Bayesian inference of population size history from multiple loci. BMC Evol Biol 8: 289. doi:10.1186/1471-2148-8-289. PubMed: 18947398. 18947398 10.1186/1471-2148-8-289 PMC2636790

[68] Rambaut A, Drummond AJ (2007) Tracer V 1: 5. Available: http://beast.bio.ed.ac.uk/Tracer.

[69] Lyngso RB, Song YS, Hein J (2005) Minimum recombination histories by branch and bound. Allgorithms Bioniformatics Proc 3692: 239-250. doi:10.1007/11557067_20.

[70] Nei M, Li WH (1979) Mathematical model for studying genetic variation in terms of restriction endonucleases. Proc Natl Acad Sci U S A 76: 5269-5273. doi:10.1073/pnas.76.10.5269. PubMed: 291943. 291943 10.1073/pnas.76.10.5269 PMC413122

[71] NIEHS (2013) SNPs NIEHS Environmental Genome Project. Seattle, WA: University of Washington.

[72] Yang Z, Bielawski JP (2000) Statistical methods for detecting molecular adaptation. Trends in Ecology &amp. Evolution 15: 496-503. 10.1016/S0169-5347(00)01994-7 PMC7134603 11114436

[73] Chimpanzee Sequencing and Analysis Consortium (2005) Initial sequence of the chimpanzee genome and comparison with the human genome. Nature 437: 69-87. doi:10.1038/nature04072. PubMed: 16136131. 16136131 10.1038/nature04072

[74] Foster CB, Aswath K, Chanock SJ, McKay HF, Peters U (2006) Polymorphism analysis of six selenoprotein genes: support for a selective sweep at the glutathione peroxidase 1 locus (3p21) in Asian populations. BMC Genet 7: 56. doi:10.1186/1471-2350-7-56. PubMed: 17156480. 17156480 10.1186/1471-2156-7-56 PMC1769511

[75] Wooding S, Kim UK, Bamshad MJ, Larsen J, Jorde LB et al. (2004) Natural selection and molecular evolution in PTC, a bitter-taste receptor gene. Am J Hum Genet 74: 637-646. doi:10.1086/383092. PubMed: 14997422. 14997422 10.1086/383092 PMC1181941

[76] Chromosome Y Consortium (2002) A nomenclature system for the tree of human Y-chromosomal binary haplogroups. Genome Res 12: 339-348. doi:10.1101/gr.217602. PubMed: 11827954. 11827954 10.1101/gr.217602 PMC155271

[77] Murisier F, Beermann F (2006) Genetics of pigment cells: lessons from the tyrosinase gene family. Histol Histopathol 21: 567-578. PubMed: 16493586. 16493586 10.14670/HH-21.567

[78] Regales L, Giraldo P, García-Díaz A, Lavado A, Montoliu L (2003) Identification and functional validation of a 5' upstream regulatory sequence in the human tyrosinase gene homologous to the locus control region of the mouse tyrosinase gene. Pigment Cell Res 16: 685-692. doi:10.1046/j.1600-0749.2003.00100.x. PubMed: 14629727. 14629727 10.1046/j.1600-0749.2003.00100.x

[79] Chen FC, Vallender EJ, Wang H, Tzeng CS, Li WH (2001) Genomic divergence between human and chimpanzee estimated from large-scale alignments of genomic sequences. J Hered 92: 481-489. doi:10.1093/jhered/92.6.481. PubMed: 11948215. 11948215 10.1093/jhered/92.6.481

[80] Scally A, Durbin R (2012) Revising the human mutation rate: implications for understanding human evolution. Nat Rev Genet 13: 745-753. doi:10.1038/nrg3295. PubMed: 22965354. 22965354 10.1038/nrg3295

[81] Blum MG, Jakobsson M (2011) Deep divergences of human gene trees and models of human origins. Mol Biol Evol 28: 889-898. doi:10.1093/molbev/msq265. PubMed: 20930054. 20930054 10.1093/molbev/msq265

[82] Garrigan D, Hammer MF (2006) Reconstructing human origins in the genomic era. Nat Rev Genet 7: 669-680. doi:10.1038/nrg1941. PubMed: 16921345. 16921345 10.1038/nrg1941

[83] Aris-Brosou S, Excoffier L (1996) The impact of population expansion and mutation rate heterogeneity on DNA sequence polymorphism. Mol Biol Evol 13: 494-504. doi:10.1093/oxfordjournals.molbev.a025610. PubMed: 8742638. 8742638 10.1093/oxfordjournals.molbev.a025610

[84] Nielsen R (2001) Statistical tests of selective neutrality in the age of genomics. Heredity (Edinb) 86: 641-647. doi:10.1046/j.1365-2540.2001.00895.x. PubMed: 11595044. 11595044 10.1046/j.1365-2540.2001.00895.x

[85] Simonsen KL, Churchill GA, Aquadro CF (1995) Properties of statistical tests of neutrality for DNA polymorphism data. Genetics 141: 413-429. PubMed: 8536987. 8536987 10.1093/genetics/141.1.413 PMC1206737

[86] Hudjashov G, Kivisild T, Underhill PA, Endicott P, Sanchez JJ et al. (2007) Revealing the prehistoric settlement of Australia by Y chromosome and mtDNA analysis. Proc Natl Acad Sci U S A 104: 8726-8730. doi:10.1073/pnas.0702928104. PubMed: 17496137. 17496137 10.1073/pnas.0702928104 PMC1885570

[87] McEvoy BP, Lind JM, Wang ET, Moyzis RK, Visscher PM et al. (2010) Whole-genome genetic diversity in a sample of Australians with deep Aboriginal ancestry. Am J Hum Genet 87: 297-305. doi:10.1016/j.ajhg.2010.07.008. PubMed: 20691402. 20691402 10.1016/j.ajhg.2010.07.008 PMC2917718

[88] Kayser M (2010) The human genetic history of Oceania: near and remote views of dispersal. Curr Biol 20: R194-R201. doi:10.1016/j.cub.2009.12.004. PubMed: 20178767. 20178767 10.1016/j.cub.2009.12.004

[89] Sabeti PC, Schaffner SF, Fry B, Lohmueller J, Varilly P et al. (2006) Positive natural selection in the human lineage. Science 312: 1614-1620. doi:10.1126/science.1124309. PubMed: 16778047. 16778047 10.1126/science.1124309

[90] Adzhubei IA, Schmidt S, Peshkin L, Ramensky VE, Gerasimova A et al. (2010) A method and server for predicting damaging missense mutations. Nat Methods 7: 248-249. doi:10.1038/nmeth0410-248. PubMed: 20354512. 20354512 10.1038/nmeth0410-248 PMC2855889

[91] Chaki M, Sengupta M, Mondal M, Bhattacharya A, Mallick S et al. (2011) Molecular and functional studies of tyrosinase variants among Indian oculocutaneous albinism type 1 patients. J Invest Dermatol 131: 260-262. doi:10.1038/jid.2010.274. PubMed: 20861851. 20861851 10.1038/jid.2010.274

[92] Tripathi RK, Hearing VJ, Urabe K, Aroca P, Spritz RA (1992) Mutational Mapping of the Catalytic Activities of Human Tyrosinase. J Biol Chem 267: 23707-23712. PubMed: 1429711. 1429711

[93] Henn BM, Botigué LR, Gravel S, Wang W, Brisbin A et al. (2012) Genomic Ancestry of North Africans Supports Back-to-Africa Migrations. PLOS Genet 8: e1002397 PubMed: 22253600. 22253600 10.1371/journal.pgen.1002397 PMC3257290

[94] Price AL, Tandon A, Patterson N, Barnes KC, Rafaels N et al. (2009) Sensitive Detection of Chromosomal Segments of Distinct Ancestry in Admixed Populations. PLOS Genet 5: e1000519 PubMed: 19543370. 19543370 10.1371/journal.pgen.1000519 PMC2689842

[95] Lu D, Willard D, Patel IR, Kadwell S, Overton L et al. (1994) Agouti protein is an antagonist of the melanocyte-stimulating-hormone receptor. Nature 371: 799-802. doi:10.1038/371799a0. PubMed: 7935841. 7935841 10.1038/371799a0

[96] Savage SA, Gerstenblith MR, Goldstein AM, Mirabello L, Fargnoli MC et al. (2008) Nucleotide diversity and population differentiation of the melanocortin 1 receptor gene, MC1R. BMC Genet 9: 31. doi:10.1186/1471-2156-9-31. PubMed: 18402696. 18402696 10.1186/1471-2156-9-31 PMC2324112

[97] 1000 Genomes Project Consortium, Abecasis GR, Altshuler D, Auton A, Brooks LD et al. (2010) A map of human genome variation from population-scale sequencing. Nature 467: 1061-1073. doi:10.1038/nature09534. PubMed: 20981092. 20981092 10.1038/nature09534 PMC3042601

[98] Grossman SR, Andersen KG, Shlyakhter I, Tabrizi S, Winnicki S et al. (2013) Identifying recent adaptations in large-scale genomic data. Cell 152: 703-713. doi:10.1016/j.cell.2013.01.035. PubMed: 23415221. 23415221 10.1016/j.cell.2013.01.035 PMC3674781

[99] Soejima M, Tachida H, Ishida T, Sano A, Koda Y (2006) Evidence for recent positive selection at the human AIM1 locus in a European population. Mol Biol Evol 23: 179-188. PubMed: 16162863. 16162863 10.1093/molbev/msj018

[100] COHMAP (1988) Climatic changes of the last 18,000 years: observations and model simulations. Science 241: 1043-1052. doi:10.1126/science.241.4869.1043. PubMed: 17747487. 17747487 10.1126/science.241.4869.1043

