# Supplementary figures and images for: Global Patterns of Diversity and Selection in Human Tyrosinase Gene
Source: PLoS One. 2013 Sep 11;8(9):e74307. doi: 10.1371/journal.pone.0074307 (PMC3770694; doi:10.1371/journal.pone.0074307)

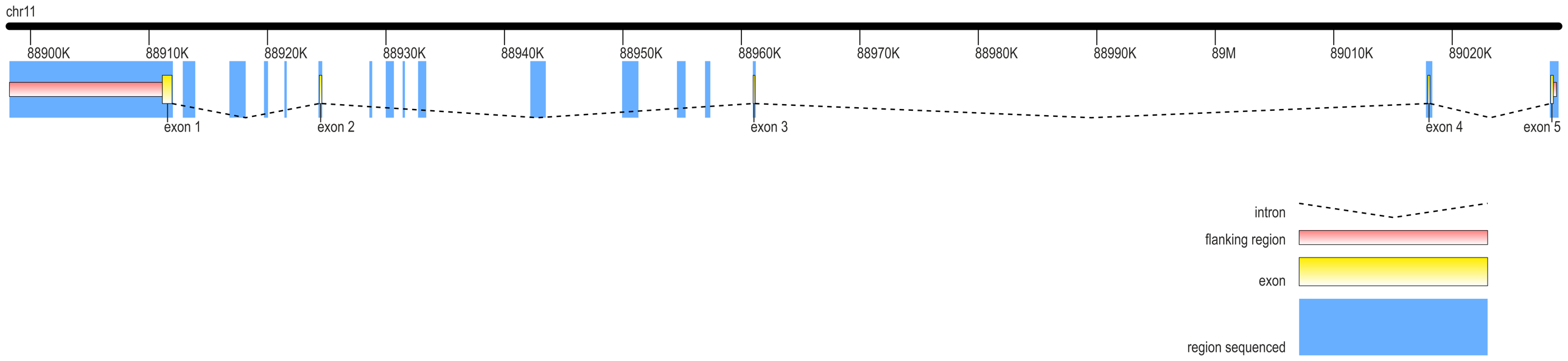

Supplement: Figure S1 — Graphical representation of TYR regions sequenced in the course of the current study. The locations of flanking regions (pink), introns (dotted line) and exons (yellow) are shown. Regions sequenced are shaded in blue. (TIF) [file pone.0074307.s001.tif]

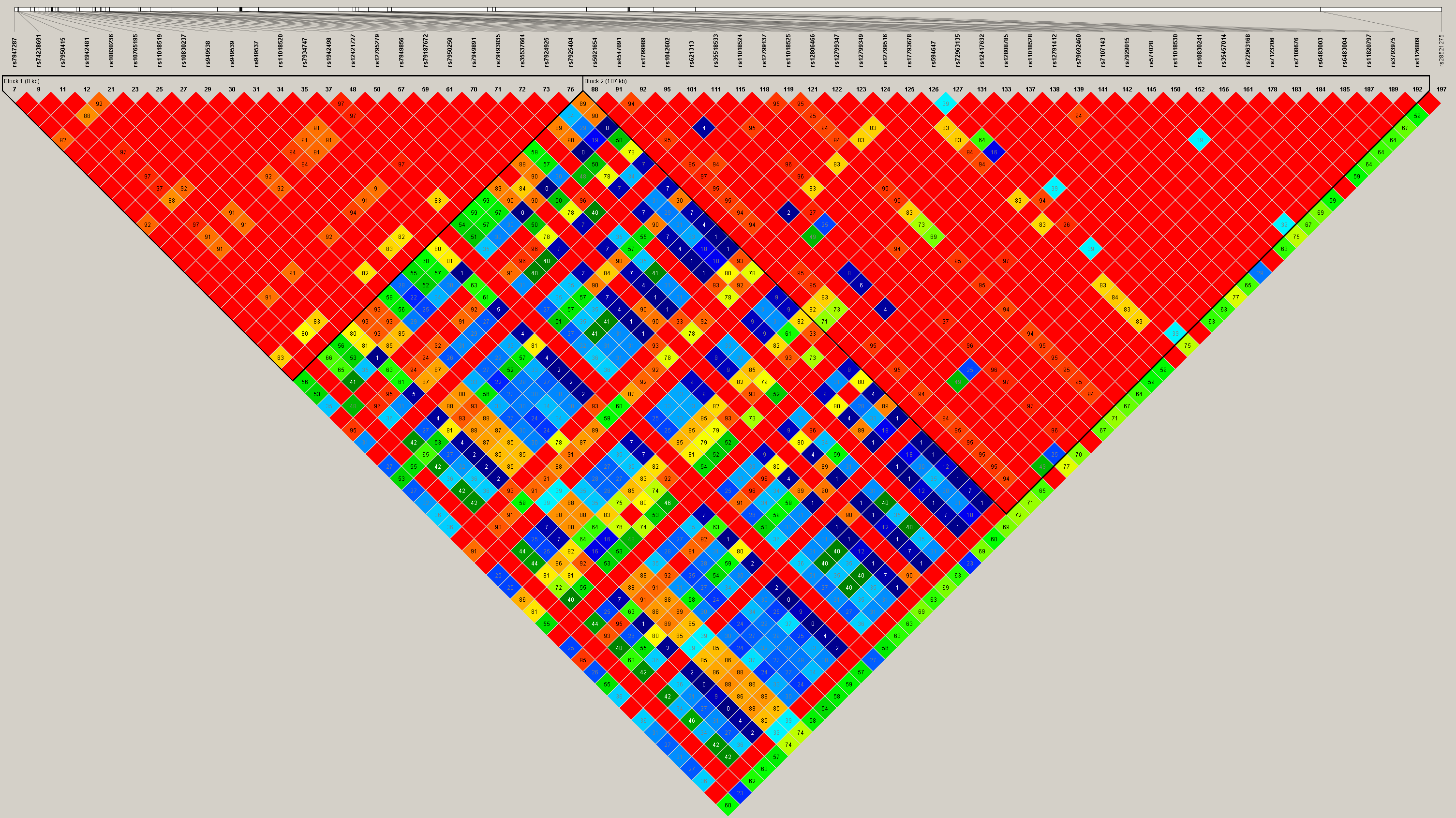

Supplement: Figure S2 — HAPLOVIEW GOLD heatmap of SNPs polymorphic in the complete re-sequencing alignment. Rare SNPs with minor allele frequency less than 0.05 are not shown. D’ values under 1 are indicated. The color scheme from blue to red indicates increasing D’ values. (TIF) [file pone.0074307.s002.tif]

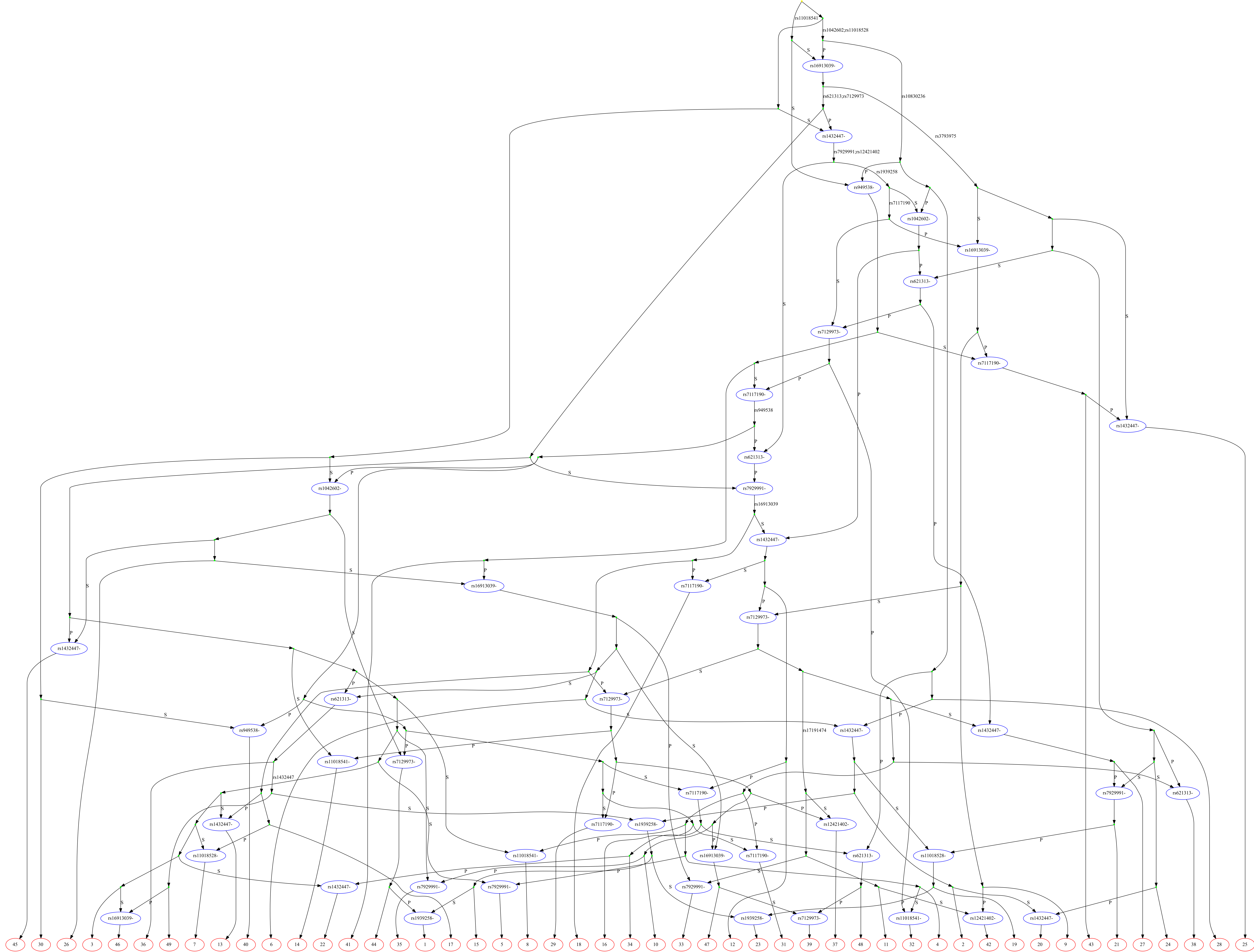[illegible]

Supplement: Figure S5 — Ancestral recombination graph showing phylogenetic relationships between 1108 samples used in the present study. The complete dataset, including samples with rare haplotypes, is shown. ARG was reconstructed using the KWARG software and represents one of the possible phylogenetic solutions of non-filtered TYR genotyping data. Haplogroup frequency by populations is shown below the tree. “P” and “S” indicate the origin of recombination prefix and suffix, respectively. Recombination points are shown in blue ovals. Individual haplotypes are shown in red ovals and coded according to Table S1. (PDF) [file pone.0074307.s005.pdf]
